# Test-retest reliability and measurement errors of grip strength test in patients with traumatic injuries in the upper extremity: a cross-sectional study

**DOI:** 10.1186/s12891-019-2623-z

**Published:** 2019-05-28

**Authors:** Zhongfei Bai, Tian Shu, Wenxin Niu

**Affiliations:** 1 Department of Occupational Therapy, Shanghai Yangzhi Rehabilitation Hospital (Shanghai Sunshine Rehabilitation Center), Shanghai, China; 2 Department of Rehabilitation Sciences, Tongji University School of Medicine, Shanghai, China

**Keywords:** Grip strength, Test-retest reliability, Measurement error, Minimal detectable change, Bland-Altman plot, Upper extremity

## Abstract

**Background:**

Grip strength (GS) test is an essential aspect of clinical practice with patients with upper extremity injuries. The random error of GS test was hypothesized to be proportional to the level of GS. The purpose of the current study was to estimate a precise range for the measurement error of GS in patients following traumatic injuries in the upper extremity.

**Methods:**

Following traumatic injuries in the upper extremity, 109 participants completed GS tests twice one weekend apart. The Bland-Altman plot analysis was adopted to estimate the precise limits of agreement with 95% confidence interval (CI).

**Results:**

The mean of three consecutive trials had a higher intraclass correlation coefficient of 0.974 (95% CI = 0.963, 0.982) than those of one trial and the mean of the first two trials in injured upper extremities. When GS was ≤20 kg, the upper limit of agreement with 95% CI was estimated as (0.41 × average GS + 1.24), while the lower limit was estimated as (− 0.41 × average GS − 0.39). A table of one-to-one matches between averaged GS ≤ 20 kg and transformed ranges of random errors with 95% certainty was created; the standard error of measurement and minimal detectable change with 95% certainty of GS test were 1.8 and 4.9 kg, respectively. When GS was > 20 kg, the width of agreement with 95% CI ranged from − 4.9 to 5.3 kg, and the standard error of measurement and minimal detectable change with 95% certainty were 1.8 and 5.1 kg, respectively.

**Conclusion:**

The one-to-one match table can be considered as a practical tool to judge a change in GS score is real or due to random errors when it is ≤20 kg.

## Background

In clinical practice, therapists concern several aspects for patients with upper extremity injuries, including pain, scarring, swelling, and range of motion of involved and adjacent joints, sensibility, muscle strength, and fine motor abilities. Among these aspects, grip strength (GS) is an essential indictor of hand function because it is a basic requirement for the performance of sports, daily activities, and work tasks [1–3]. Additionally, GS can also reflect general health status and, more specifically, it is negatively associated with cardiovascular mortality, myocardial infarction, and stroke [4]. Therefore, reliable GS measures are important for evaluating the severity of a disability and for monitoring clinical progress.

The intraclass correlation coefficient (ICC) is traditionally used to estimate the agreement between two repeated administrations [5, 6]. Previous studies related to the measurement properties of GS showed that hand dynamometer has satisfactory test-retest reliability in upper extremities with physical dysfunction [7–9]. To determine patients’ changes in a specific measurement are real or due to random errors, the minimal detectable change with 95% certainty (MDC_95_) is used as a parameter to estimate the size of random errors [10–12]. Therefore, by knowing the MDC of GS in patients with upper extremity injuries, clinicians can determine the change in GS score is likely to be the result of a real improvement or caused by random measurement errors. Schreuders et al. [8] estimated the test-retest reliability of GS test in patients with hand injuries and reported an ICC of 0.97, with an MDC_95_ of 61 N (≈ 6.22 kg). This shows that differences between two consecutive measurements greater than 61 N can be interpreted as real changes in GS, with 95% certainty. Nevertheless, in clinical practice with patients with upper extremity injuries, many patients at sub-acute stage may experience a very low GS score of only a few kilograms. To the present authors’ knowledge, the MDC_95_ of 61 N may be too large for patients with only a few kilograms of GS, and we consider that it is quite impossible for patients with extremely poor GS to have such relatively large random errors. Although clinicians may have high confidence in determining if patients’ changes are real when GS scores are greater than the large MDC_95_, it will result in high possibility of false-negative interpretations.

The Bland-Altman plot complements the role of ICC and MDC in determining test-retest reliability of measurement tools. The plot, usually presented as differences of two measurements against the mean of two measurements, can reveal the 95% limits of agreement (LoA_95_), which is the width of the differences with 95% certainty. The LoA_95_ defines a range within which most differences will lie, and a narrow range of LoA_95_ indicates that the scores of two measurements are close together [13]. Whether and how a relationship exists between them can be identified through statistical analysis and visual inspection [13]. In a study evaluating the test-retest reliability of the Jamar Dynamometer in a healthy population of 76 participants, the Bland-Altman plot seemed to indicate that the differences were proportional to the mean [14]. In addition, we found similar scatters in the Bland-Altman plot of another study with 19 healthy participants [15]. Therefore, we hypothesized that the random errors between two administrations are also proportional to GS in patients with upper extremity injuries. Furthermore, it is not appropriate to determine the change in GS score is real or due to random errors by using MDC_95_ alone in patients with different levels of GS ranging from several kilograms to tens of kilograms.

The purpose of this study was to estimate the test-retest reliability and the precise range of measurement errors of GS test in patients with upper extremity injuries using the Bland-Altman plot analysis to help clinical practitioners to determine that patients’ changes in GS indicate real progress or are due to random errors.

## Methods

### Design

This research was a clinical measurement and cross-sectional study. Participants received GS tests twice; more specifically, the first test was administered on Friday and we carried out the second test on the following Monday.

### Participants

Patients with upper extremity dysfunction due to traumatic occupational injuries were recruited in a rehabilitation center. All patients were receiving inpatient rehabilitation services in the rehabilitation center when they were recruited. The following inclusion criteria were applied: (1) aged 18 years or above; (2) having a traumatic injury in unilateral upper extremity; (3) being capable of being evaluated for GS, confirmed by an occupational therapist experienced in hand therapy; (4) remaining dysfunction in injured upper extremities; and (5) having good compliance with occupational therapists’ daily treatment instructions. The following exclusion criteria were applied: (1) having concurrent injuries in any other parts of the body; (2) experiencing pain when performing maximal isometric GS (visual analogue scale > 3); and (3) not being able to attend the second GS test.

All participants signed an informed consent form in accordance with the Declaration of Helsinki, and the study was approved by the medical ethics committee of the rehabilitation center.

### Procedures

In this rehabilitation center, all patients receive rehabilitation services five days per week, from Monday to Friday. During weekends, they usually go home or stay in wards and do not receive any formal rehabilitation services from clinical practitioners. The aim of this study was to estimate the test-retest reliability and the range of measurement errors of GS test. To avoid any bias from interventions, we arranged the first test on Friday and the second test on the following Monday. Therefore, we hypothesized that because no effective interventions were delivered in the short interval between the two tests, none of the participants would have experienced a real change in GS. After signing the consent form, demographic data including gender, marital status, age, height, body weight, and dominant hand were collected from each participant. In addition, injured sides, injury sites, and the number of days since injuries were confirmed.

### GS test

Prior to starting the first test, participants were instructed to sit on a chair and maintain the posture recommended by the American Society of Hand Therapy [16] and Roberts et al. [17]. The participants sat with their feet flat on the floor, the shoulder adducted 0 degree, the elbow flexed at 90 degrees, the forearm in a neutral position, and the wrist extended to 30 degrees. The dynamometer used in this study was a calibrated Jamar Hydraulic Hand Dynamometer (model SH5001, Saehan Corp, Masan, Korea) which was the most commonly used one and showed excellent reliability for the measurement of GS in previous studies [17]. Verbal instructions and demonstration about how to perform GS test were provided to each participant prior to the test. Once everything was ready, the participants were instructed to exert maximum grip at the second handle position and to maintain the contraction for five seconds. Three consecutive trials were performed with both injured and healthy upper extremities and there was 15 s of rest period among trials to prevent muscle fatigue. All participants started the test with their healthy hands. The value at which the needle of the dynamometer stopped was recorded for each trial. The second test followed the above procedures and used the same dynamometer for all patients. In the current study, the same occupational therapist experienced in hand therapy was responsible for all participants’ GS tests.

### Statistical analysis

Descriptive statistics were computed to illustrate participants’ demographic characteristics. Both the one-sample Kolmogorov-Smirnov test and histogram plot were applied to check for the normality of continuous variables. We used the data of the first trial, the mean of the first two trials (mean_2_), and the mean of the three trials (mean_3_) to estimate the test-retest reliability and the measurement error of GS of injured and healthy upper extremities. ICC_2,1_ as well as their 95% confidence intervals (CI) were calculated [5]. An ICC value higher than 0.9 was considered excellent. In addition, a paired *t*-test was applied to verify if there was any systematic bias between the first and second tests. The MDC_95_ and standard error of measurement (SEM) were calculated using the following formulas [18]:

1 $$ {\mathrm{MDC}}_{95}=1.96\times \sqrt{2}\times \mathrm{SEM} $$

2 $$ \mathrm{SEM}=\mathrm{SD}\times \sqrt{1- ICC} $$

To verify whether there were any other relationships between GS and measurement errors, the Bland-Altman plots were created based on the values of mean_3_. A systematic error is confirmed if the 95% CI for the mean value of differences does not include 0. The LoA_95_ was calculated by using the Bland-Altman plots which present the scatter of differences between the first and second tests (y-axis) against the average of the first and second GS tests (average GS) (x-axis) [19]. If the differences are normally distributed and do not show any associations with the average GS, limits of the LoA_95_ are computed as

3 $$ {\mathrm{LoA}}_{95}={\mathrm{mean}}_{\mathrm{difference}}\pm 1.96\ {\mathrm{SD}}_{\mathrm{difference}} $$

where mean_difference_ is the mean of differences between the two tests, and SD_difference_ is the standard deviation of the differences. This implies that 95% of the differences will lie between the upper and lower limits.

In injured upper extremities, the Spearman’s correlation coefficient *ρ* between the observed differences, which were not normally distributed, and the average GS was 0.118 (*p* = 0.310). Therefore, residuals were defined as the differences between observed differences and the mean of differences. It was observed that the absolute values of residuals (|*R*|), which were the distances between the observed differences and mean_difference_, tended to increase as the average GS increased in upper extremities with poor GS. However, in upper extremities with high GS, this trend was not distinct. To identify the most appropriate cutoff point on the average GS to separate the above two conditions, the Spearman’s correlation coefficient *ρ* between the |*R*| and the average GS lower than each possible cutoff point on the average GS was calculated. This was because the |*R*| was not normally distributed. The cutoff was defined as the point where the relationship between the |*R*| and the average GS had the highest Spearman’s correlation coefficient. The Bland-Altman plots were then constructed again for the two conditions according to Bland and Altman’s recommendations [13]. First, we regressed the |*R*| on the average GS to derive

4 $$ \left|R\right|=c0+c1\times \mathrm{average}\ \mathrm{GS} $$

Second, the LoA_95_ was calculated using the following formula:

5 $$ {\mathrm{LoA}}_{95}={\mathrm{mean}}_{\mathrm{difference}}\pm 1.96\times \sqrt{\pi \div 2}\times \mid R\mid $$

Once upper and lower limits of the LoA_95_ were calculated, one-to-one matches between integral GS scores and transformed ranges of random errors with 95% certainty were created for convenience in clinical application. The transformed lower and upper limits of the ranges of random errors were calculated using the integral GS scores plus the upper and lower limits of LoA_95_, respectively.

All statistical analyses were performed with the IBM SPSS Statistics 20. The level of significance was set at *p* < 0.05 for all statistical analyses performed.

## Results

### Sample characteristics

A total of 111 patients participated in the current study between February and September 2017, of whom two patients did not attend the second test. Therefore, 109 patients were included in the statistical analysis, and their median age was 37 years. In terms of the types of injuries, 52 (47.7%) patients had fractures, followed by 18 (16.5%), 14 (12.8%), 14 (12.8%), and 11 (10.1%) for finger replantation, finger amputation, complex injuries involving tendons, and complex injuries involving nerves, respectively. The median days since being injured was 133. Characteristics of the participants are presented in full in Table 1.

**Table 1:** Characteristics of included participants

Participant demographics (*n* = 109)	*n* (%)
Age in years, median (lower quartile–upper quartile)	37 (27–46)
Height in centimetres, median (lower quartile–upper quartile)	168 (162–173)
Weight in kilograms, median (lower quartile–upper quartile)	63 (57–72)
Gender	
Male	74 (67.9%)
Female	35 (32.1%)
Marital status	
Married	93 (85.3%)
Single	16 (14.7%)
Days since injury, median (lower quartile–upper quartile)	133 (85–227)
Injury side	
Dominant	54 (49.5%)
Non-dominant	55 (50.5%)
Injury sites	
Hand	68 (62.4%)
Wrist	16 (14.7%)
Forearm	9 (8.3%)
Shoulder	5 (4.6%)
Elbow	8 (7.3%)
Upper arm	3 (2.8%)
Types of injuries	
Fracture	52 (47.7%)
Finger replantation	18 (16.5%)
Finger amputation	14 (12.8%)
Complex injuries involved tendons	14 (12.8%)
Complex injuries involved nerves	11 (10.1%)

### The test-retest reliability of GS test

The indices of test-retest reliability of GS test in injured and healthy upper extremities based on the data of the first trial, mean_2_, and mean_3_ are presented in Table 2. No significant differences were observed between the first and second test scores, which ranged from 0.1 (95% CI = − 0.7, 0.8) kg to 0.7 (95% CI = 0, 1.4) kg. The test-retest reliabilities of GS in injured and healthy upper extremities were excellent, with high ICCs ranging from 0.936 (95% CI = 0.908, 0.956) to 0.974 (95% CI = 0.963, 0.982). Although mean_3_ had the highest ICC of 0.956 (95% CI = 0.936, 0.970) in healthy upper extremities, it did not differ significantly from those of mean_2_ and the first trial, 0.949 (95% CI = 0.926, 0.965) and 0.936 (95% CI = 0.908, 0.956), respectively. In contrast, in injured upper extremities, mean_3_ had the highest ICC of 0.974 (95% CI = 0.963, 0.982), which was significantly higher than that of the first trial, 0.945 (95% CI = 0.920, 0.962). However, the ICC of mean_3_ was not significantly different from that of mean_2_, 0.970 (95% CI = 0.956, 0.979). Mean_3_ had the lowest SEM and MDC_95_, 1.8 kg and 5.0 kg, respectively, compared with mean_2_ and the first trial in injured upper extremities (Table 2).

**Table 2:** Reliability indices of grip strength test

Grip strength	*n*	The first test	The second test	Difference		Paired *t*	ICC^a^ (95% CI)	SEM^b^	MDC_95_^c^
		mean ± *SD* (kg)	mean ± *SD* (kg)	mean ± *SD* (kg)	mean (95% CI) (kg)	*P*-value			
First trial (healthy)	109	35.4 ± 10.7	35.4 ± 10.4	0.1 ± 3.8	0.1 (−0.7, 0.8)	0.880	0.936 (0.908, 0.956)	2.7	7.4
Mean_2_^d^ (healthy)	109	34.6 ± 10.2	34.8 ± 10.1	0.2 ± 3.3	0.2 (−0.4, 0.8)	0.503	0.949 (0.926, 0.965)	2.3	6.4
Mean_3_^e^ (healthy)	109	34.1 ± 9.9	34.3 ± 9.9	0.2 ± 3.0	0.2 (−0.4, 0.7)	0.601	0.956 (0.936, 0.970)	2.1	5.8
First trial (injured)	109	15.0 ± 11.5	15.8 ± 11.6	0.7 ± 3.8	0.7 (0.0, 1.4)	0.052	0.945 (0.920, 0.962)	2.7	7.4
Mean_2_ (injured)	109	15.1 ± 11.2	15.4 ± 11.4	0.4 ± 2.8	0.4 (−0.1, 0.9)	0.140	0.970 (0.956, 0.979)	2.0	5.4
Mean_3_ (injured)	109	15.1 ± 11.2	15.4 ± 11.3	0.4 ± 2.5	0.4 (−0.1, 0.8)	0.143	0.974 (0.963, 0.982)	1.8	5.0
Mean_3_ (injured, ≤ 20 kg)	76	8.9 ± 5.7	9.3 ± 6.1	0.4 ± 2.5	0.4 (−0.2, 1.0)	0.145	0.908 (0.859, 0.941)	1.8	4.9
Mean_3_ (injured, > 20 kg)	33	29.3 ± 6.6	29.5 ± 6.9	0.2 ± 2.6	0.2 (−0.7, 1.1)	0.659	0.928 (0.859, 0.964)	1.8	5.1

^a^Intraclass correlation coefficient

^b^Standard error measurement

^c^Minimal detectable change with 95% certainty

^d^Mean of the first two trials

^e^Mean of the three trials

### The Bland-Altman plot analysis based on mean_3_

**Fig. 1:**
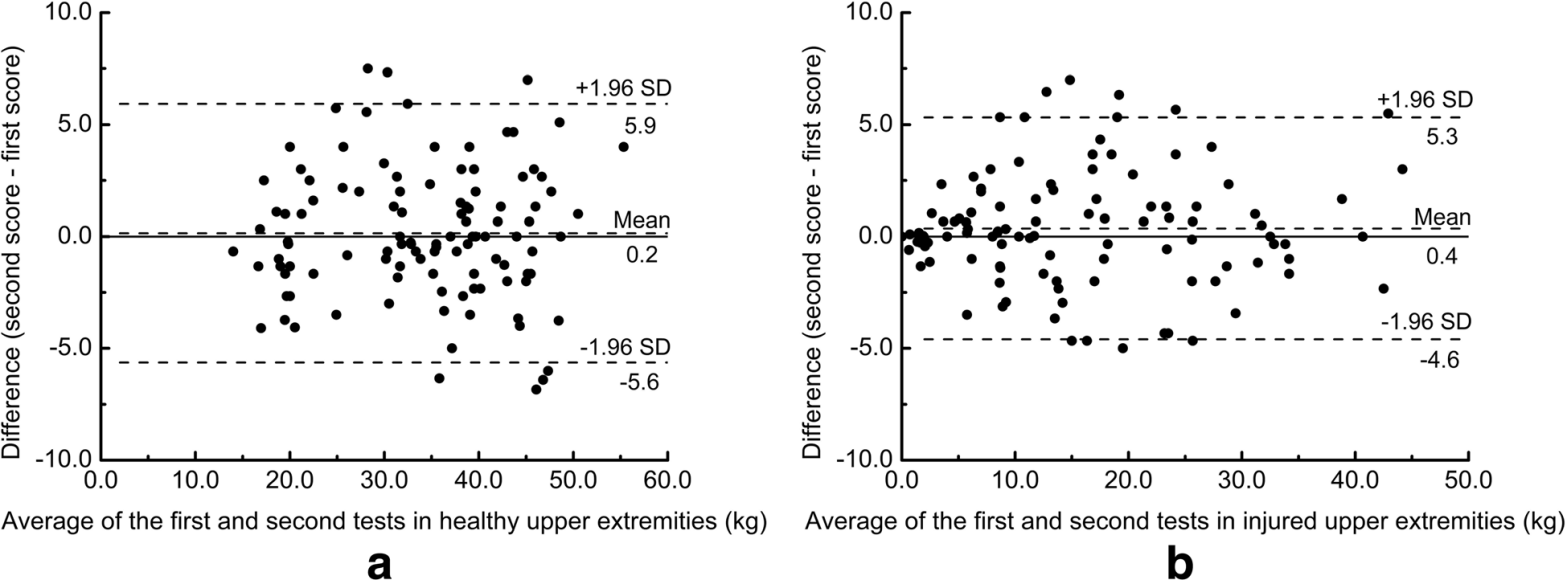
Bland-Altman plots for the test-retest reliability of the hand grip test based on the mean of three trials in (**a**) healthy and (**b**) injured upper extremities

The Bland-Altman plot analysis was conducted for further examination of the differences. In healthy upper extremities, the Bland-Altman plot showed no systematic trend (Fig. 1 a). The mean difference between the second and first tests was 0.2 (95% CI = − 0.4, 0.7) kg. The width of LoA_95_ was − 5.6 to 5.9 kg, and 101 (92.7%) cases fell within the 95% limits of agreement. In injured upper extremities, the mean difference between the second and first tests was 0.4 (95% CI = − 0.1, 0.8) kg. The width of LoA_95_ was − 4.6 to 5.3 kg (Fig. 1 b), and 97 (89.0%) cases fell within the 95% limits of agreement. Visual inspection suggested a trend whereby the absolute value of residuals seemed to be proportional to the value along the average GS in injured upper extremities with poor GS. However, in injured upper extremities with high GS, this trend was not distinct.

**Fig. 2:**
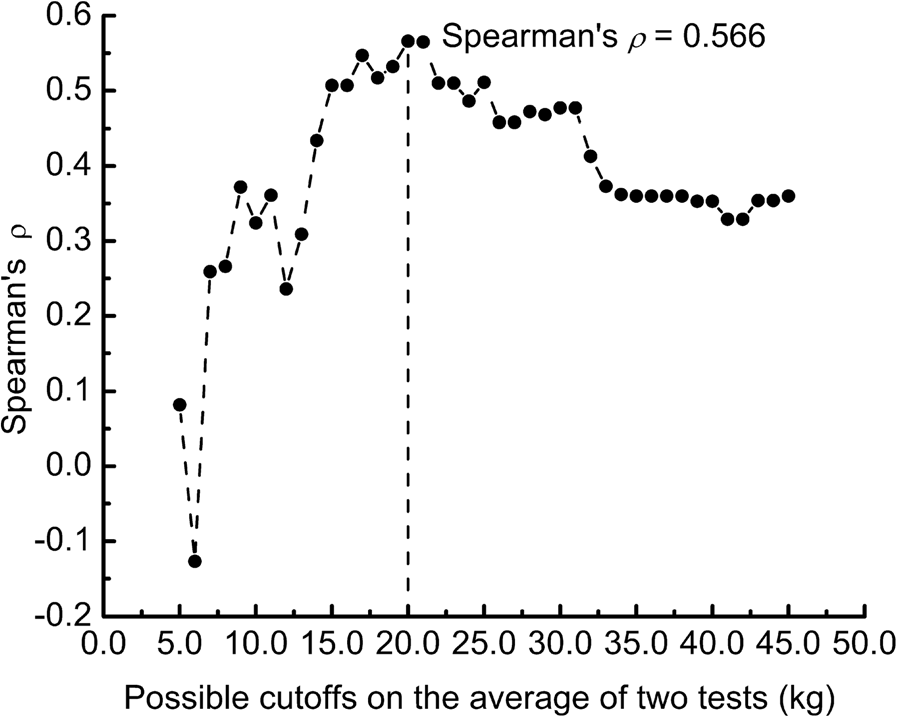
Values of Spearman’s correlation coefficient *ρ* between the absolute values of residuals and average grip strength of two tests lower than possible cutoffs

The Spearman’s correlation coefficients between residuals and average GS scores lower than possible cutoffs were calculated (Fig. 2). The results indicated that 20 kg was the most appropriate cutoff with the highest correlation coefficient (Spearman’s *ρ* = 0.566, *p* < 0.001). The Spearman’s *ρ* between the absolute values of residuals and the average GS in injured upper extremities with GS ≤ 20 was 0.566 (95% CI = 0.406, 0.896, *p* < 0,001), but − 0.003 (95% CI = − 0.352, 0.355, *p* = 0.987) in injured upper extremities with GS >  20 kg. Findings for the test-retest reliability and measurement errors based on mean_3_ ≤ 20 kg and >  20 kg in injured upper extremities are presented in Table 2. For injured upper extremities with GS ≤ 20 kg (*n* = 76), the SEM and MDC_95_ were 1.8 kg and 4.9 kg, respectively; while the SEM and MDC_95_ were 1.8 kg and 5.1 kg in injured upper extremities with GS > 20 kg (*n* = 33), respectively.

**Fig. 3:**
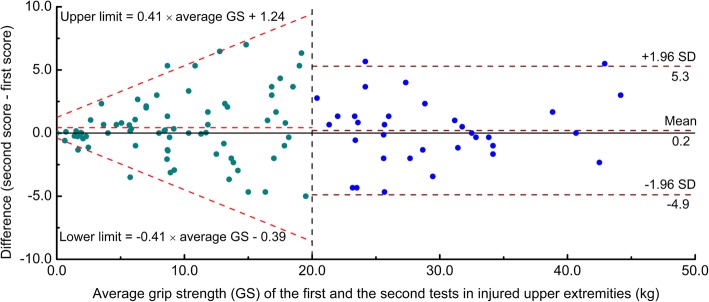
The Bland-Altman plots for the test-retest reliability of the hand grip test in injured upper extremities with the mean of three trials ≤20 kg and > 20 kg

As shown in Fig. 3, for injured upper extremities with average GS ≤ 20 kg, the Bland-Altman plot showed a narrow LoA_95_ in upper extremities with poor GS, but the LoA_95_ width increased as the average GS increased. The mean difference between the second and first tests was 0.43 (95% CI = − 0.15, 1.00). The limits of the agreement estimated were:

6 $$ \mathrm{Upper}\ \mathrm{limit}=0.41\times \mathrm{average}\ \mathrm{GS}+1.24 $$

7 $$ \mathrm{Lower}\ \mathrm{limit}=-0.41\times \mathrm{average}\ \mathrm{GS}-0.39 $$

One-to-one matches between GS scores ≤20 kg and ranges of random errors with 95% certainty were created and are presented in Table 3.

**Table 3:** One-to-one matches between grip strength and transformed ranges of random errors with 95% certainty when the grip strength is ≤20 kg

Grip strength	Lower limit	Upper limit
1.0	0.2	2.7
2.0	0.8	4.0
3.0	1.4	5.5
4.0	2.0	6.9
5.0	2.6	8.3
6.0	3.2	9.7
7.0	3.7	11.1
8.0	4.3	12.5
9.0	4.9	13.9
10.0	5.5	15.3
11.0	6.1	16.8
12.0	6.7	18.2
13.0	7.3	19.6
14.0	7.9	21.0
15.0	8.5	22.4
16.0	9.1	23.8
17.0	9.6	25.2
18.0	10.2	26.6
19.0	10.8	28.0
20.0	11.4	29.4

In injured upper extremities with GS scores > 20 kg, the Bland-Altman plot based on mean_3_ showed no systematic difference between the first and second tests and no distinct trend between residuals and average GS scores. The mean difference between the second and first tests was 0.2 (95% CI = − 0.7, 1.1) kg, and the width of LoA_95_ was − 4.9 to 5.3 kg. In total, 104 (95.4%) cases fell within the LoA_95_ in the new Bland-Altman plot analyses.

## Discussion

The inter-rater reliability of GS test has been shown excellent in previous study [20]. The current study estimated the test-retest reliability of GS test based on results of the first trial, the mean of the first two trials, and the mean of three consecutive trials, and calculated the SEM as well as MDC. In the current study, Bland-Altman plot analysis was adopted to explore the relationship between measurement errors and GS in healthy and injured upper extremities.

In healthy upper extremities, our findings were consistent with previous studies, which evaluated the test-retest reliability of hand-held dynamometers [21–25]. Our study confirmed that the GS test using Jamar Dynamometer had excellent reliability and was not affected by practice effect. In addition, although mean_3_ had the highest ICC, it was not significantly different from those of the first trial and mean_2_. This indicated that these three methods had comparable reliability and supported the one-trial protocol for assessing GS in healthy upper extremities [26]. Visual inspection of the Bland-Altman plot for healthy upper extremities did not show signs of any systematic bias in the relationships between differences and GS scores. Therefore, the MDC_95_ could be considered as an ideal criterion to determine that the changes in GS of healthy upper extremities are real or due to random error.

However, in injured upper extremities, mean_3_ had a significantly higher ICC than that of the first trial, but its difference from mean_2_ was non-significant. The SEM and MDC_95_ of mean_3_ were also the lowest in injured upper extremities. In particular, the paired *t*-test for the first trial of injured upper extremities showed a *p*-value close to the significance threshold, and the lower limit of 95% CI for the mean difference was zero. This revealed that there might be a systematic bias which influenced the reliability. Kennedy et al. [27] found that both one trial and mean_3_ had comparable test-retest reliability based on a sample of 25 participants with rheumatoid arthritis. However, we recruited 109 participants in the current study, which resulted in narrow 95% CIs for the ICC. Therefore, the ICC values of the first trial (ICC = 0.945) and mean_3_ (ICC = 0.974) did not exhibit a large difference, but it was significantly different. Accordingly, we considered that the first trial, mean_2_, and mean_3_ had excellent test-retest reliability, among which the mean_3_ method was the most reliable. Therefore, we support the use of the mean_3_ method to test patients’ GS in clinical practice, as recommended by the American Society of Hand Therapy [16], even though it would entail extra time for the test.

The Bland-Altman plot is a graphical method to identify any relationships between the differences and averages of scores on two tests [13]. In our study, a trend was observed whereby the difference was proportional to the average of two GS tests in injured upper extremities with poor GS. However, the width of the differences in injured upper extremities with high GS was stable. We used the Spearman’s correlation coefficient to identify the ideal cutoff point where the relationship between the absolute values of residuals and average GS had the highest Spearman’s correlation coefficient. Our findings showed that 20 kg was the most appropriate cutoff point to separate injured upper extremities into the above two conditions. Additionally, we estimated the width of LoA_95_ for injured upper extremities with GS ≤ 20 kg according to the recommendations of Bland and Altman [13]. The graph of LoA_95_ looked like a “horn,” which indicated that the measurement error increased as GS increased when GS was ≤20 kg. To take the example of a GS score of 5 kg, the width of the measurement error was − 3.5 to 3.4 kg, according to the horn-like LoA_95_. However, according to the MDC_95_, the width of the measurement error was − 4.9 to 4.9 kg. Therefore, when the MDC_95_ was used to determine whether a patient’s change was beyond the threshold of random error, the possibility of a false-negative interpretation would be increased.

To simplify clinical application of the equations we proposed, we transformed them into a table giving one-to-one matches between GS scores and ranges of random errors with 95% certainty. For the clinical application of this table, clinicians can first find their patients’ current level of GS in the left column of the table, and then the lower and upper limits of the corresponding range of random errors can be determined. Specifically, a patient’s change may not be real if the result of the second test lies within the corresponding range. In addition, upon combining the Bland-Altman plot for GS ≤ 20 and GS > 20 kg, a higher percentage of cases was found to fall within the 95% limits of agreement.

Our study may be the first one with a large sample size which constructed the Bland-Altman plots for poor and high GS scores. In the past, many authors adopted the Bland-Altman plots to analyze the reliability of GS test, but they did not conduct the analysis presented in our study. The most important reason might be their small sample sizes [15, 28, 29]. Scatter plots of small sample sizes usually cannot easily indicate obvious relationships, and statistical analyses may easily yield non-significant results. Therefore, such analyses were ignored in previous studies. In our study, the Spearman’s *ρ* between the absolute values of residuals and the average of the first and second tests was 0.566 in 76 patients with GS ≤ 20 kg, and post hoc analysis showed a statistical power of 0.999, indicating a powerful statistical significance. On the other hand, previous researchers commonly evaluated the reliability of devices for GS test in healthy populations showing high level of GS score [14]. However, our study revealed that the GS score of injured upper extremities ranged from several kilograms to tens of kilograms, which covered the full range of GS scores. This was another reason why we could identify the relationship between measurement errors and GS scores.

This study also had some limitations. Firstly, the present participants received rehabilitation services on weekdays. We hypothesized that they did not undergo any real change over the weekend because they did not receive any formal interventions during this period. However, two confounding factors might have influenced the results of the current study: the lasting effect of interventions received during weekdays and additional exercises done by the participants during the weekend. Generally speaking, the lasting effect and additional exercises could improve participants’ GS scores and increase the extent of disagreement between the two tests. This may be the reason why the paired *t*-test for the first trial of injured upper extremities showed a *p*-value close to the significance level. Secondly, only 33 participants had GS score > 20 kg. Therefore, we could not make conclusions with strong confidence as to whether the appropriate cutoff point had changed if we recruited more participants with GS > 20 kg. Thirdly, to avoid any learning effect, some researchers employed a warm-up practice prior to GS test in addition to verbal instructions and demonstration [30, 31]. However, in the current study, we provide verbal instructions and demonstration only and this may have a negative influence on the reliability of GS test. Lastly, we only sampled participants who had traumatic injuries and only used one commercial hand-hold dynamometer to estimate the measurement error in the current study. Therefore, we cannot be certain that our results can be generalized to other disorders and devices to asses GS.

## Conclusions

In summary, the GS test was found to have excellent test-retest reliability in healthy and injured upper extremities. We also recommend that clinical practitioners should use mean_3_ for GS test, particularly in cases with injured upper extremities. When the GS is ≤20 kg, clinicians can use the one-to-one match table to judge a change in GS is real or due to random errors.

## Abbreviations

|*R*|: Absolute values of residuals
CI: Confidence intervals
GS: Grip strength
ICC: Intraclass correlation coefficient
LoA_95_: 95% limits of agreement
MDC_95_: Measurement minimal detectable change with 95% certainty
Mean_2_: Mean of the first two trials
Mean_3_: Mean of the three trials
SD: Standard deviation
SEM: Standard error of measurement
